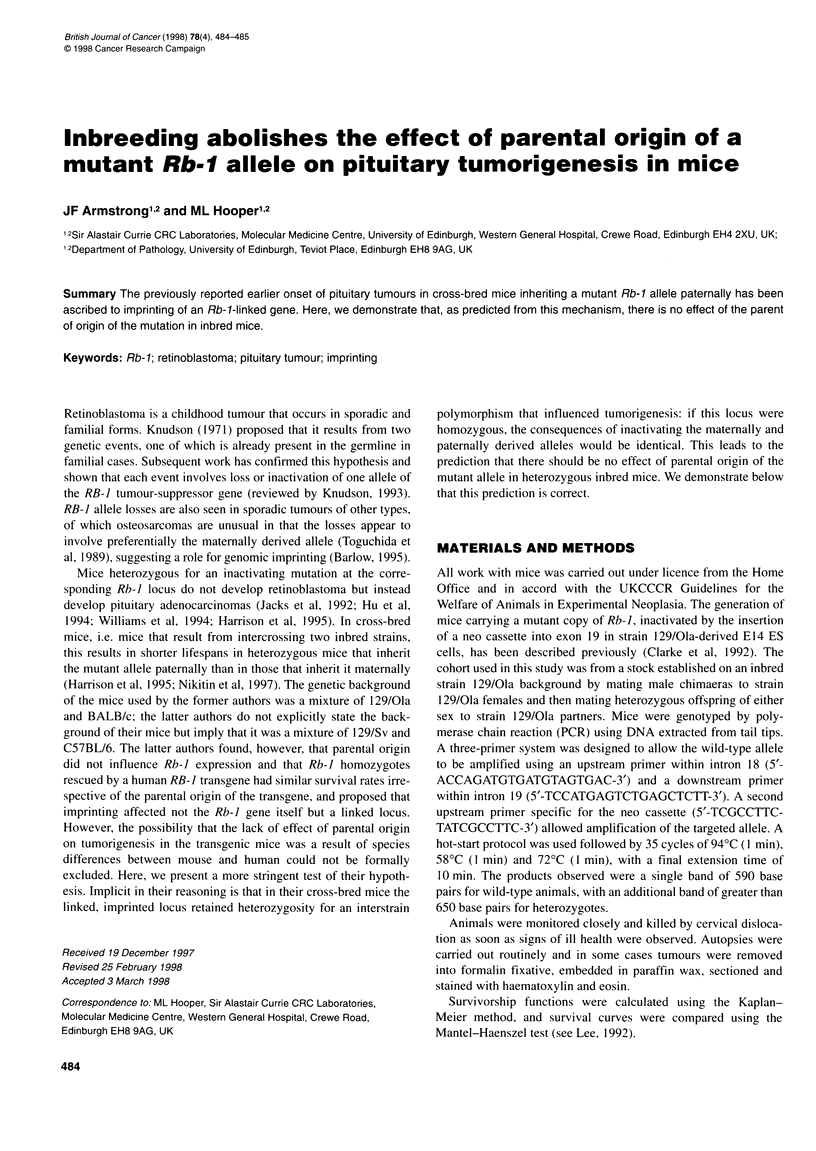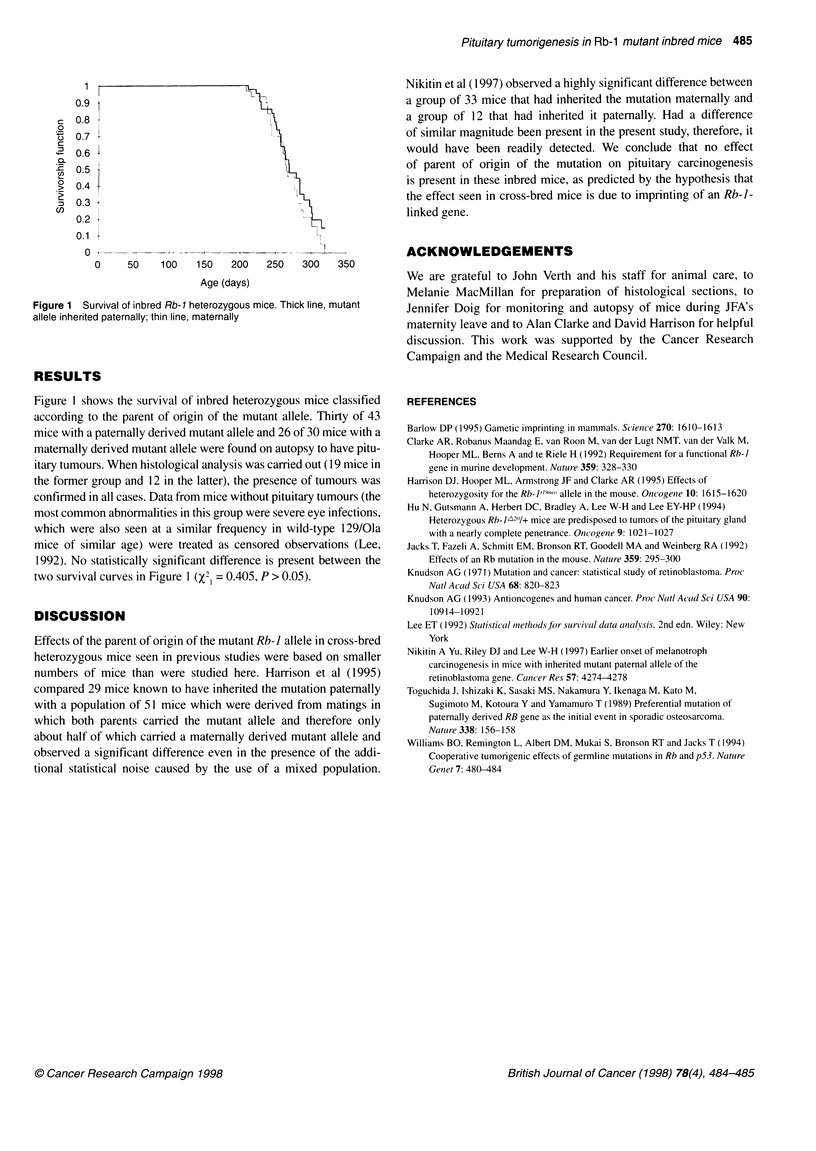# Inbreeding abolishes the effect of parental origin of a mutant Rb-1 allele on pituitary tumorigenesis in mice.

**DOI:** 10.1038/bjc.1998.519

**Published:** 1998-08

**Authors:** J. F. Armstrong, M. L. Hooper

**Affiliations:** Sir Alastair Currie CRC Laboratories, Molecular Medicine Centre, University of Edinburgh, Western General Hospital, UK.

## Abstract

The previously reported earlier onset of pituitary tumours in cross-bred mice inheriting a mutant Rb-1 allele paternally has been ascribed to imprinting of an Rb-1-linked gene. Here, we demonstrate that, as predicted from this mechanism, there is no effect of the parent of origin of the mutation in inbred mice.


British Journal of Cancer (1998) 78(4), 484–485
© 1998 Cancer Research Campaign

### Inbreeding abolishes the effect of parental origin of a mutant *Rb-1* allele on pituitary tumorigenesis in mice

**JF Armstrong[1,2] and ML Hooper[1,2]**

[1,2]Sir Alastair Currie CRC Laboratories, Molecular Medicine Centre, University of Edinburgh, Western General Hospital, Crewe Road, Edinburgh EH4 2XU, UK; [1,2]Department of Pathology, University of Edinburgh, Teviot Place, Edinburgh EH8 9AG, UK

**Summary** The previously reported earlier onset of pituitary tumours in cross-bred mice inheriting a mutant *Rb-1* allele paternally has been ascribed to imprinting of an *Rb-1*-linked gene. Here, we demonstrate that, as predicted from this mechanism, there is no effect of the parent of origin of the mutation in inbred mice.

**Keywords:** *Rb-1*; retinoblastoma; pituitary tumour; imprinting

Retinoblastoma is a childhood tumour that occurs in sporadic and familial forms. Knudson (1971) proposed that it results from two genetic events, one of which is already present in the germline in familial cases. Subsequent work has confirmed this hypothesis and shown that each event involves loss or inactivation of one allele of the *RB-1* tumour-suppressor gene (reviewed by Knudson, 1993). *RB-1* allele losses are also seen in sporadic tumours of other types, of which osteosarcomas are unusual in that the losses appear to involve preferentially the maternally derived allele (Toguchida et al, 1989), suggesting a role for genomic imprinting (Barlow, 1995).

Mice heterozygous for an inactivating mutation at the corresponding *Rb-1* locus do not develop retinoblastoma but instead develop pituitary adenocarcinomas (Jacks et al, 1992; Hu et al, 1994; Williams et al, 1994; Harrison et al, 1995). In cross-bred mice, i.e. mice that result from intercrossing two inbred strains, this results in shorter lifespans in heterozygous mice that inherit the mutant allele paternally than in those that inherit it maternally (Harrison et al, 1995; Nikitin et al, 1997). The genetic background of the mice used by the former authors was a mixture of 129/Ola and BALB/c; the latter authors do not explicitly state the background of their mice but imply that it was a mixture of 129/Sv and C57BL/6. The latter authors found, however, that parental origin did not influence *Rb-1* expression and that *Rb-1* homozygotes rescued by a human *RB-1* transgene had similar survival rates irrespective of the parental origin of the transgene, and proposed that imprinting affected not the *Rb-1* gene itself but a linked locus. However, the possibility that the lack of effect of parental origin on tumorigenesis in the transgenic mice was a result of species differences between mouse and human could not be formally excluded. Here, we present a more stringent test of their hypothesis. Implicit in their reasoning is that in their cross-bred mice the linked, imprinted locus retained heterozygosity for an interstrain polymorphism that influenced tumorigenesis: if this locus were homozygous, the consequences of inactivating the maternally and paternally derived alleles would be identical. This leads to the prediction that there should be no effect of parental origin of the mutant allele in heterozygous inbred mice. We demonstrate below that this prediction is correct.

*Received 19 December 1997*
*Revised 25 February 1998*
*Accepted 3 March 1998*

*Correspondence to:* ML Hooper, Sir Alastair Currie CRC Laboratories, Molecular Medicine Centre, Western General Hospital, Crewe Road, Edinburgh EH8 9AG, UK

### MATERIALS AND METHODS

All work with mice was carried out under licence from the Home Office and in accord with the UKCCCR Guidelines for the Welfare of Animals in Experimental Neoplasia. The generation of mice carrying a mutant copy of *Rb-1*, inactivated by the insertion of a neo cassette into exon 19 in strain 129/Ola-derived E14 ES cells, has been described previously (Clarke et al, 1992). The cohort used in this study was from a stock established on an inbred strain 129/Ola background by mating male chimaeras to strain 129/Ola females and then mating heterozygous offspring of either sex to strain 129/Ola partners. Mice were genotyped by polymerase chain reaction (PCR) using DNA extracted from tail tips. A three-primer system was designed to allow the wild-type allele to be amplified using an upstream primer within intron 18 (5′-ACCAGATGTGATGTAGTGAC-3′) and a downstream primer within intron 19 (5′-TCCATGAGTCTGAGCTCTT-3′). A second upstream primer specific for the neo cassette (5′-TCGCCTTCTATCGCCTTC-3′) allowed amplification of the targeted allele. A hot-start protocol was used followed by 35 cycles of 94°C (1 min), 58°C (1 min) and 72°C (1 min), with a final extension time of 10 min. The products observed were a single band of 590 base pairs for wild-type animals, with an additional band of greater than 650 base pairs for heterozygotes.

Animals were monitored closely and killed by cervical dislocation as soon as signs of ill health were observed. Autopsies were carried out routinely and in some cases tumours were removed into formalin fixative, embedded in paraffin wax, sectioned and stained with haematoxylin and eosin.

Survivorship functions were calculated using the Kaplan–Meier method, and survival curves were compared using the Mantel–Haenszel test (see Lee, 1992).

**Figure 1** Survival of inbred *Rb-1* heterozygous mice. Thick line, mutant allele inherited paternally; thin line, maternally

### RESULTS

Figure 1 shows the survival of inbred heterozygous mice classified according to the parent of origin of the mutant allele. Thirty of 43 mice with a paternally derived mutant allele and 26 of 30 mice with a maternally derived mutant allele were found on autopsy to have pituitary tumours. When histological analysis was carried out (19 mice in the former group and 12 in the latter), the presence of tumours was confirmed in all cases. Data from mice without pituitary tumours (the most common abnormalities in this group were severe eye infections, which were also seen at a similar frequency in wild-type 129/Ola mice of similar age) were treated as censored observations (Lee, 1992). No statistically significant difference is present between the two survival curves in Figure 1 ($\chi^2_1 = 0.405$, $P > 0.05$).

### DISCUSSION

Effects of the parent of origin of the mutant *Rb-1* allele in cross-bred heterozygous mice seen in previous studies were based on smaller numbers of mice than were studied here. Harrison et al (1995) compared 29 mice known to have inherited the mutation paternally with a population of 51 mice which were derived from matings in which both parents carried the mutant allele and therefore only about half of which carried a maternally derived mutant allele and observed a significant difference even in the presence of the additional statistical noise caused by the use of a mixed population. Nikitin et al (1997) observed a highly significant difference between a group of 33 mice that had inherited the mutation maternally and a group of 12 that had inherited it paternally. Had a difference of similar magnitude been present in the present study, therefore, it would have been readily detected. We conclude that no effect of parent of origin of the mutation on pituitary carcinogenesis is present in these inbred mice, as predicted by the hypothesis that the effect seen in cross-bred mice is due to imprinting of an *Rb-1*-linked gene.

### ACKNOWLEDGEMENTS

We are grateful to John Verth and his staff for animal care, to Melanie MacMillan for preparation of histological sections, to Jennifer Doig for monitoring and autopsy of mice during JFA's maternity leave and to Alan Clarke and David Harrison for helpful discussion. This work was supported by the Cancer Research Campaign and the Medical Research Council.

### REFERENCES

Barlow DP (1995) Gametic imprinting in mammals. *Science* **270**: 1610–1613

Clarke AR, Robanus Maandag E, van Roon M, van der Lugt NMT, van der Valk M, Hooper ML, Berns A and te Riele H (1992) Requirement for a functional *Rb-1* gene in murine development. *Nature* **359**: 328–330

Harrison DJ, Hooper ML, Armstrong JF and Clarke AR (1995) Effects of heterozygosity for the $Rb\text{-}1^{t19neo}$ allele in the mouse. *Oncogene* **10**: 1615–1620

Hu N, Gutsmann A, Herbert DC, Bradley A, Lee W-H and Lee EY-HP (1994) Heterozygous $Rb\text{-}1^{\Delta 20}/+$ mice are predisposed to tumors of the pituitary gland with a nearly complete penetrance. *Oncogene* **9**: 1021–1027

Jacks T, Fazeli A, Schmitt EM, Bronson RT, Goodell MA and Weinberg RA (1992) Effects of an Rb mutation in the mouse. *Nature* **359**: 295–300

Knudson AG (1971) Mutation and cancer: statistical study of retinoblastoma. *Proc Natl Acad Sci USA* **68**: 820–823

Knudson AG (1993) Antioncogenes and human cancer. *Proc Natl Acad Sci USA* **90**: 10914–10921

Lee ET (1992) *Statistical methods for survival data analysis*, 2nd edn. Wiley: New York

Nikitin A Yu, Riley DJ and Lee W-H (1997) Earlier onset of melanotroph carcinogenesis in mice with inherited mutant paternal allele of the retinoblastoma gene. *Cancer Res* **57**: 4274–4278

Toguchida J, Ishizaki K, Sasaki MS, Nakamura Y, Ikenaga M, Kato M, Sugimoto M, Kotoura Y and Yamamuro T (1989) Preferential mutation of paternally derived *RB* gene as the initial event in sporadic osteosarcoma. *Nature* **338**: 156–158

Williams BO, Remington L, Albert DM, Mukai S, Bronson RT and Jacks T (1994) Cooperative tumorigenic effects of germline mutations in *Rb* and *p53*. *Nature Genet* **7**: 480–484

© Cancer Research Campaign 1998